# The effect of sex on the efficacy and safety of dual antithrombotic therapy with dabigatran versus triple therapy with warfarin after PCI in patients with atrial fibrillation (a RE‐DUAL PCI subgroup analysis and comparison to other dual antithrombotic therapy trials)

**DOI:** 10.1002/clc.23649

**Published:** 2021-05-27

**Authors:** David S. Eccleston, Joseph M. Kim, Jurien M. ten Berg, P. Gabriel Steg, Deepak L. Bhatt, Stefan H. Hohnloser, Anne de Veer, Matias Nordaby, Corinna Miede, Takeshi Kimura, Gregory Y. H. Lip, Jonas Oldgren, Christopher P. Cannon

**Affiliations:** ^1^ Department of Medicine University of Melbourne and GenesisCare Melbourne Australia; ^2^ Brigham and Women's Hospital and Harvard Medical School Boston Massachusetts USA; ^3^ Department of Cardiology St. Antonius Ziekenhuis Nieuwegein Netherlands; ^4^ FACT, an F‐CRIN Network, DHU FIRE Université Paris Diderot, INSERM U_1148 and Hôpital Bichat Assistance Publique Paris France; ^5^ Johann Wolfgang Goethe University Department of Cardiology, Division of Clinical Electrophysiology Frankfurt am Main Germany; ^6^ Boehringer Ingelheim GmbH Ingelheim Germany; ^7^ Mainanalytics GmbH Sulzbach (Taunus) Germany; ^8^ Kyoto University Department of Cardiovascular Medicine Kyoto Japan; ^9^ Liverpool Centre for Cardiovascular Science University of Liverpool and Liverpool Heart & Chest Hospital Liverpool UK; ^10^ Department of Clinical Medicine, Aalborg Thrombosis Research Unit Aalborg University Aalborg Denmark; ^11^ Department of Medical Sciences and Uppsala Clinical Research Center Uppsala University Uppsala Sweden

**Keywords:** dual antithrombotic therapy, sex differences, triple therapy

## Abstract

**Background:**

The RE‐DUAL PCI trial demonstrated that in patients with nonvalvular atrial fibrillation (AF) undergoing percutaneous coronary intervention (PCI), dual therapy with dabigatran and a P2Y_12_ inhibitor, either clopidogrel or ticagrelor, reduced the risk of bleeding without an increased risk of thromboembolic events as compared to triple therapy with warfarin in addition to a P2Y_12_ inhibitor and aspirin. What remains unclear is whether this effect is consistent between males and females undergoing PCI.

**Hypothesis:**

The reduction in risk of bleeding without increased risk of thromboembolic events with dual therapy with dabigatran and a P2Y_12_ inhibitor in comparison to triple therapy with warfarin, a P2Y_12_ inhibitor and aspirin is consistent in females and males.

**Methods:**

The primary safety endpoint was the first International Society on Thrombosis and Hemostasis (ISTH) major bleeding event (MBE) or clinically relevant non‐major bleeding event (CRNMBE). The efficacy endpoint was the composite of death, thromboembolic event (stroke, myocardial infarction, and systemic embolism) or unplanned revascularization. Cox proportional hazard regression analyses were applied to calculate corresponding hazard ratios and interaction p values for each endpoint.

**Results:**

A total of 655 women and 2070 men were enrolled. The risk of major or CRNM bleeding was lower with both dabigatran 110 mg dual therapy and dabigatran 150 mg dual therapy compared with warfarin triple therapy in female and male patients (for 110 mg: females: HR 0.69, 95% CI 0.47–1.01, males: HR 0.46, 95% CI 0.37–0.59, interaction p value: 0.084 and for 150 mg: females HR 0.74, 95% CI 0.48–1.16, males HR 0.71, 95% CI 0.56–0.90, interaction p value: 0.83). There was also no detectable difference in the composite efficacy endpoint of death, thromboembolic events or unplanned revascularization between dabigatran dual therapy and warfarin triple therapy, with no statistically significant interaction between sex and treatment (interaction p values: 0.73 and 0.72, respectively).

**Conclusions:**

Consistent with the overall study results, the risk of bleeding was lower with dabigatran 110 mg and 150 mg dual therapy compared with warfarin triple therapy, and risk of thromboembolic events was comparable with warfarin triple therapy independent of the patient's sex.

## 1 INTRODUCTION

Sex differences in treatments and outcomes are increasingly recognized in medicine, particularly in cardiovascular medicine. Previous studies suggest females are underrepresented in clinical trials, with less female‐specific data in terms of drug safety and efficacy.^1^, ^2^ Women with heart disease including atrial fibrillation are older and have more co‐morbidities, and the potential benefit–risk of new therapies for both adverse cardiovascular events as well as bleeding may differ.^3^, ^4^, ^5^, ^6^, ^7^, ^8^, ^9^, ^10^ For patients with atrial fibrillation who undergo percutaneous coronary intervention (PCI), this balance is particularly important – with high risk of thrombotic events and bleeding.

The Randomized Evaluation of Dual Antithrombotic Therapy with Dabigatran versus Triple Therapy with Warfarin in Patients with Nonvalvular Atrial Fibrillation Undergoing Percutaneous Coronary Intervention (RE‐DUAL PCI) trial assessed the safety and efficacy of dabigatran dual therapy at doses of 110 or 150 mg with a P2Y_12_ inhibitor (either clopidogrel or ticagrelor) versus warfarin plus aspirin (ASA) and a P2Y_12_ inhibitor (either clopidogrel or ticagrelor) in patients with AF undergoing PCI. We assessed whether outcomes seen in RE‐DUAL PCI are generalizable and applicable to females. We then compared these results to those of four other trials comparing triple therapy to dual therapy so as to gain a broader perspective and allow tailoring of therapy to the specific needs of females.

## 2 METHODS

### 2.1 Trial design and treatment

The design and the results of the RE‐DUAL PCI trial (NCT02164864) have been reported previously.^11^, ^12^ Briefly, 2725 patients were randomly assigned to receive dual therapy with dabigatran 110 or 150 mg twice daily plus either clopidogrel or ticagrelor, or to receive triple therapy with warfarin (adjusted to achieve an international normalized ratio of 2.0–3.0) plus ASA (≤100 mg daily) and either clopidogrel or ticagrelor. Outside the United States, patients aged ≥80 years (≥70 years in Japan) were randomized only to the 110 mg dabigatran dose versus warfarin in order to be consistent with the labeling of dabigatran in elderly patients in non‐US countries. The study was approved by each center's institutional review board and all patients provided written informed consent.

All patients received clopidogrel (75 mg daily) or ticagrelor (90 mg twice daily) for ≥12 months after randomization. In the warfarin triple therapy group, ASA was discontinued after 1 month in patients implanted with a bare‐metal stent (BMS) and after 3 months in patients with a drug‐eluting stent (DES).

### 2.2 Patients

Inclusion criteria, as detailed previously, included males and females aged ≥18 years with non‐valvular AF who underwent PCI with a BMS or DES within the previous 120 h.^11^, ^12^ The indication for PCI included both acute coronary syndrome (ACS) or stable coronary artery disease. Exclusion criteria included patients with bioprosthetic or mechanical heart valves, severe renal insufficiency (creatinine clearance <30 ml/min at screening, calculated by the Cockcroft‐Gault equation) or other major coexisting conditions.

### 2.3 Assessments

The primary endpoint was the time to a first International Society on Thrombosis and Hemostasis (ISTH) major bleeding events (MBEs) or clinically relevant non‐major bleeding events (CRNMBEs) during follow‐up (mean 14 months). Additionally, we evaluated the rates of Thrombolysis in Myocardial Infarction (TIMI) major and minor bleeds and intracranial hemorrhage (ICH) events. The time to the composite endpoint of death, thromboembolic events (myocardial infarction, stroke or systemic embolism), or unplanned revascularization (first event) was evaluated, and the individual rates of MI, definite stent thrombosis, stroke, CV death and all‐cause death, respectively.

### 2.4 Statistical analysis

For the comparison of dabigatran 110 mg dual therapy versus warfarin triple therapy within the two subgroup categories (male vs female), stratified Cox proportional hazard regression models were applied, which included age as a stratifying factor (non‐elderly vs elderly ages: <70 vs ≥70 years in Japan and <80 vs ≥80 years elsewhere) and treatment arm. For the dabigatran 150 mg dual therapy versus warfarin triple therapy comparison, corresponding unstratified Cox proportional hazard regression models were applied (excluding patients aged ≥80 years [≥70 years in Japan] outside the United States). Corresponding hazard ratios (HRs) and two‐sided 95% Wald confidence intervals (CIs) for HRs were calculated for each subgroup category. Exploratory treatment by subgroup interaction p values resulting from Cox proportional hazard regression models were provided. Bleeding and thromboembolic outcomes were assessed according to treatment group and by sex (female vs male).

### 2.5 Comparison across trials

The assessments in the primary endpoint of bleeding and the main ischemic/thromboembolic endpoint by sex as seen in RE‐DUAL PCI was compared with those same endpoints in four other trials: WOEST, PIONEER AF‐PCI, ENTRUST‐AF PCI, and AUGUSTUS.^13^, ^14^, ^15^, ^16^ Individual trial definitions of bleeding and ischemia were used. Hazard ratios, 95% CIs, and interaction p values delineated by sex were presented in the trial results in all but WOEST and ENTRUST‐AF PCI for which the data was provided by Prof. Jurrien ten Berg and Prof. Pascal Vranckx, respectively.

## 3 RESULTS

### 3.1 Patient characteristics

A total of 2725 patients were randomized; 2070 patients were male (76.0%) and 655 (24.0%) were female. Overall females were older at time of PCI than males (73.2 ± 7.9 vs 70.0 ± 8.8 years). By definition, the mean CHA_2_DS_2_‐VASc scores were higher in females than in males (4.5 versus 3.3). The HAS‐BLED scores were similar in females than in males (2.8 vs 2.7) (Table 1). Of the 327 patients who received ticagrelor, 74 were female (22.6%) and of the 2363 patients who received clopidogrel, 577 were female (24.4%).

**TABLE 1:** Baseline characteristics of patients

	Females		Males		Females		Males	
	Dabigatran 110 mg Dual Therapy (*n* = 253)	Warfarin Triple Therapy (*n* = 231)	Dabigatran 110 mg Dual Therapy (*n* = 728)	Warfarin Triple Therapy (*n* = 750)	Dabigatran 150 mg Dual Therapy (*n* = 171)	Warfarin Triple Therapy^d^ (*n* = 170)	Dabigatran 150 mg Dual Therapy (*n* = 592)	Warfarin Triple Therapy^d^ (*n* = 594)
Mean age, years (*SD*)	74.3 (8.2)	74.2 (7.5)	70.6 (8.9)	71.0 (9.2)	70.5 (7.2)	71.1 (6.1)	68.1 (7.7)	68.2 (8.0)
AF at baseline, *n* (%)^a^								
Paroxysmal	134 (53.0)	117 (50.6)	353 (48.5)	367 (48.9)	91 (53.2)	85 (50.0)	289 (48.8)	291 (49.0)
Persistent	40 (15.8)	39 (16.9)	134 (18.4)	139 (18.5)	29 (17.0)	35 (20.6)	103 (17.4)	114 (19.2)
Permanent	79 (31.2)	75 (32.5)	241 (33.1)	243 (32.4)	51 (29.8)	50 (29.4)	199 (33.6)	188 (31.6)
Diabetes, *n* (%)^a^	114 (45.1)	93 (40.3)	248 (34.1)	278 (37.1)	66 (38.6)	75 (44.1)	194 (32.8)	228 (38.4)
Prior Stroke, *n* (%)^a^	17 (6.7)	19 (8.2)	57 (7.8)	81 (10.8)	18 (10.5)	14 (8.2)	34 (5.7)	63 (10.6)
Prior MI, *n* (%)	47 (18.6)	39 (16.9)	190 (26.1)	229 (30.5)	30 (17.5)	27 (15.9)	164 (27.7)	184 (31.0)
Mean CrCl, ml/min (*SD*)^b^	65.1 (25.4)	64.6 (22.0)	80.1 (29.0)	78.6 (30.1)	73.8 (31.0)	68.4 (23.0)	86.5 (30.4)	84.9 (30.2)
Mean CHA_2_DS_2_‐VASc (*SD*)	4.6 (1.3)	4.6 (1.3)	3.4 (1.5)	3.5 (1.5)	4.3 (1.4)	4.5 (1.4)	3.0 (1.4)	3.3 (1.5)
Mean HAS‐BLED (*SD*)	2.8 (0.56)	2.9 (0.68)	2.7 (0.72)	2.7 (0.75)	2.7 (0.65)	2.9 (0.68)	2.6 (0.71)	2.7 (0.77)
OAC Treatment At Baseline, *n* (%)								
Long‐term	89 (35.2)	62 (26.8)	258 (35.4)	273 (36.4)	49 (28.7)	48 (28.2)	198 (33.4)	196 (33.0)
Treatment‐naïve	164 (64.8)	169 (73.2)	470 (64.6)	477 (63.6)	122 (71.3)	122 (71.8)	394 (66.6)	398 (67.0)
Stent type, *n* (%)^c^								
DES only	204 (80.6)	187 (81.0)	600 (82.4)	639 (85.2)	131 (76.6)	138 (81.2)	490 (82.8)	500 (84.2)
BMS only	41 (16.2)	41 (17.7)	107 (14.7)	92 (12.3)	33 (19.3)	30 (17.6)	90 (15.2)	77 (13.0)
DES + BMS and other	7 (2.8)	2 (0.9)	20 (2.7)	15 (2.0)	6 (3.5)	1 (0.6)	12 (2.0)	13 (2.2)

Abbreviations: AF, atrial fibrillation; BMS, bare‐metal stent; DES, drug‐eluting stent; MI, myocardial infarction; OAC, oral anticoagulant; *SD*, standard deviation.

^a^ Data missing from one male.

^b^ Data missing from 63 females and 169 males,

^c^ Data missing from three females and five males.

^d^ For the comparison with dabigatran 150 mg dual therapy, elderly patients outside the United States are excluded.

### 3.2 Bleeding events

**FIGURE 1:**
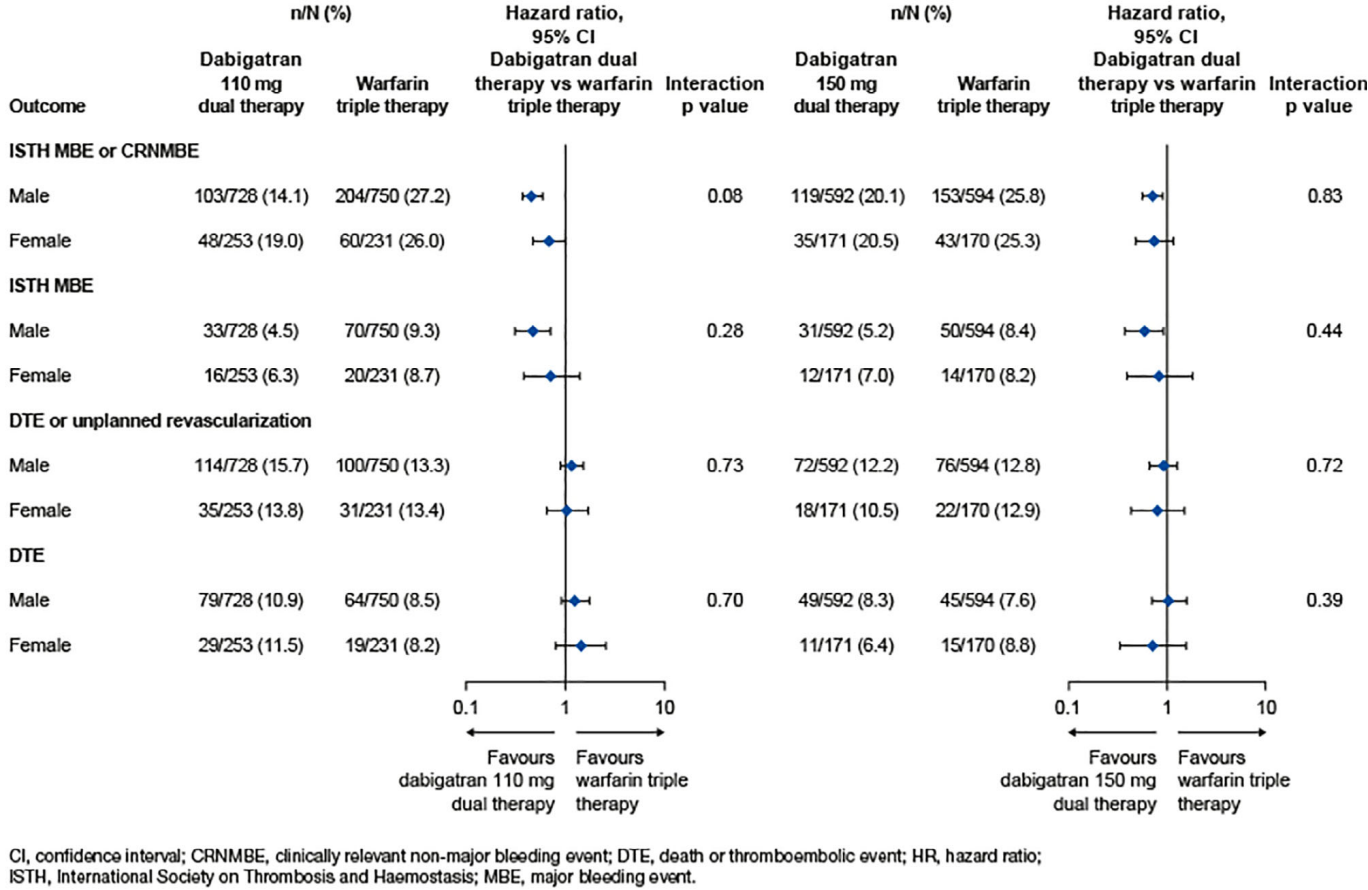
The risk of bleeding and major ischemic events (death and thromboembolic events) by patient sex. HR and 95% CIs from Cox proportional hazard regression model stratified by age (elderly vs non‐elderly) for dabigatran 110 mg dual therapy versus warfarin triple therapy, and unstratified for dabigatran 150 mg dual therapy versus warfarin triple therapy. For the comparison with dabigatran 150 mg dual therapy, elderly patients outside the United States are excluded. CI, confidence interval; CRNMBE, clinically relevant nonmajor bleeding event; DE, dabigatran etexilate; DTE, death or thromboembolic event; HR, hazard ratio; ISTH, International Society on Thrombosis and Hemostasis; MBE, major bleeding event

Looking at the event rates, females tended to have higher rates of ISTH MBE or CRNMBE compared to males, a finding seen in other CV trials.^4^, ^17^, ^18^ Treatment effects of dual therapy with dabigatran dosed at 110 mg versus warfarin triple therapy were consistent for females and males for this endpoint (females: HR 0.69, 95% CI 0.47–1.01, males: HR 0.46, 95% CI 0.37–0.59, interaction p value: 0.08) (Figure 1). Consistent treatment effects were also seen for the primary endpoint with dual therapy with dabigatran dosed at 150 mg vs triple therapy with warfarin in female and male patients (females HR 0.74, 95% CI 0.48–1.16, males HR 0.71, 95% CI 0.56–0.90, interaction p value: 0.83). In the evaluation of the total bleeds, ISTH MBE and CRNMBE, Thrombolysis in Myocardial Infarction (TIMI) major bleeds, TIMI minor bleeds, and intracranial hemorrhage (ICH) individually, no statistically significant interaction between sex and treatment for both 110 mg and 150 mg of dabigatran dual therapy versus warfarin triple therapy could be detected (Table 2).

**TABLE 2:** Bleeding and thromboembolic events by sex

Bleeding events									
	Females				Males				Interaction p value
	Dabigatran 110 mg dual therapy (%) (*n* = 253)	Warfarin triple therapy (%) (*n* = 231)	Hazard ratio	95% CI	Dabigatran 110 mg dual therapy (%) (*n* = 728)	Warfarin triple therapy (%) (*n* = 750)	Hazard ratio	95% CI	
Total bleed	77 (30.4)	94 (40.7)	0.68	0.50–0.92	189 (26.0)	327 (43.6)	0.50	0.42–0.60	0.10
ISTH MBE	16 (6.3)	20 (8.7)	0.72	0.38–1.40	33 (4.5)	70 (9.3)	0.47	0.31–0.71	0.28
CRNMBE	34 (13.4)	47 (20.3)	0.62	0.40–0.96	81 (11.1)	146 (19.5)	0.52	0.40–0.69	0.53
TIMI Major Bleed	1 (0.4)	8 (3.5)	0.12	0.01–0.92	13 (1.8)	29 (3.9)	0.45	0.23–0.87	0.21
TIMI Minor Bleed	7 (2.8)	9 (3.9)	0.70	0.26–1.87	8 (1.1)	26 (3.5)	0.31	0.14–0.69	0.22
ICH	1 (0.4)	4 (1.7)	0.23	0.03–2.07	2 (0.3)	6 (0.8)	0.33	0.07–1.66	0.79

	Females				Males				Interaction p value
	Dabigatran 150 mg dual therapy (%) (*n* = 171)	Warfarin triple therapy (%) (*n* = 170)	Hazard ratio	95% CI	Dabigatran 150 mg dual therapy (%) (*n* = 592)	Warfarin triple therapy (%) (*n* = 594)	Hazard ratio	95% CI	
Total bleed	58 (33.9)	64 (37.6)	0.84	0.59–1.20	196 (33.1)	252 (42.4)	0.68	0.57–0.82	0.30
ISTH MBE	12 (7.0)	14 (8.2)	0.84	0.39–1.81	31 (5.2)	50 (8.4)	0.59	0.37–0.92	0.44
CRNMBE	28 (16.4)	35 (20.6)	0.75	0.45–1.23	98 (16.6)	113 (19.0)	0.81	0.62–1.06	0.77
TIMI Major Bleed	3 (1.8)	7 (4.1)	0.42	0.11–1.62	13 (2.2)	23 (3.9)	0.54	0.27–1.06	0.75
TIMI Minor Bleed	5 (2.9)	4 (2.4)	1.20	0.32–4.48	7 (1.2)	17 (2.9)	0.40	0.16–0.96	0.16
ICH	0 (0.0)	3 (1.8)	NA	NA	1 (0.2)	5 (0.8)	0.20	0.02–1.67	0.99

Thromboembolic events									
	Females				Males				Interaction p value
	Dabigatran 110 mg dual therapy (%) (*n* = 253)	Warfarin triple therapy (%) (*n* = 231)	Hazard ratio	95% CI	Dabigatran 110 mg dual therapy (%) (*n* = 728)	Warfarin triple therapy (%) (*n* = 750)	Hazard ratio	95% CI	
All cause death	14 (5.5)	11 (4.9)	1.18	0.54–2.60	41 (5.6)	37 (4.9)	1.11	0.71–1.73	0.90
CV death	8 (3.2)	6 (2.6)	1.23	0.43–3.56	29 (4.0)	25 (3.3)	1.16	0.68–1.97	0.90
MI	12 (4.7)	7 (3.0)	1.60	0.63–4.06	32 (4.4)	22 (2.9)	1.48	0.86–2.55	0.90
Stent thrombosis	5 (2.0)	3 (1.3)	1.60	0.38–6.71	10 (1.4)	5 (0.7)	2.02	0.69–5.92	0.79
Stroke	7 (2.8)	2 (0.9)	3.16	0.66–15.21	10 (1.4)	11 (1.5)	0.93	0.39–2.18	0.17

	Females				Males				Interaction p value
	Dabigatran 150 mg dual therapy (%) (*n* = 171)	Warfarin triple therapy (%) (*n* = 170)	Hazard ratio	95% CI	Dabigatran 150 mg dual therapy (%) (*n* = 592)	Warfarin triple therapy (%) (*n* = 594)	Hazard ratio	95% CI	
All cause death	7 (4.1)	8 (4.7)	0.86	0.31–2.38	23 (3.9)	27 (4.5)	0.81	0.47–1.42	0.91
CV death	4 (2.3)	5 (2.9)	0.79	0.21–2.96	17 (2.9)	19 (3.2)	0.85	0.44–1.64	0.93
MI	5 (2.9)	6 (3.5)	0.81	0.25–2.65	21 (3.5)	16 (2.7)	1.29	0.67–2.47	0.50
Stent thrombosis	1 (0.6)	3 (1.8)	0.33	0.03–3.16	6 (1.0)	4 (0.7)	1.48	0.42–5.25	0.25
Stroke	2 (1.2)	2 (1.2)	1.00	0.14–7.11	7 (1.2)	6 (1.0)	1.12	0.38–3.34	0.91

Abbreviations: CV, cardiovascular; CRNMBE, clinically relevant non‐major bleeding event; ICH, intracranial hemorrhage; ISTH, international society on thrombosis and hemostasis; NA, one of the categories had an n of 0 in which a hazard ratio is not generated; TIMI: thrombolysis in myocardial infarction.

*Note*: HR and 95% CIs from Cox proportional hazard regression model stratified by age (elderly vs non‐ elderly) for dabigatran 110 mg dual therapy versus warfarin triple therapy, and unstratified for dabigatran 150 mg dual therapy versus warfarin triple therapy. For the comparison with dabigatran 150 mg dual therapy, elderly patients outside the United States are excluded.

### 3.3 Thromboembolic events

No interaction between sex and treatment was observed for dual therapy with dabigatran at either the 110 mg or 150 mg doses vs triple therapy with warfarin with regard to the composite efficacy endpoint of death, thromboembolic events or unplanned revascularization (interaction p values: 0.73 and 0.72). When MI, stent thrombosis, stroke, CV death and all cause death were evaluated individually, no statistically significant interaction between treatment and sex were observed in any of these outcomes with either the 110 mg or 150 mg doses of dabigatran dual therapy versus warfarin triple therapy although the numbers of events for many of these individual endpoints is small which limits its interpretation. (Table 2).

### 3.4 Comparison across trials

There were considerably fewer females than males included in WOEST, PIONEER AF‐PCI, ENTRUST‐AF PCI, and AUGUSTUS, with each trial enrolling ~25% females across all trials. Similar to the results from RE‐DUAL PCI, the results from WOEST, PIONEER AF‐PCI, ENTRUST‐AF PCI, and AUGUSTUS showed no interaction between sex and treatment for neither the bleeding endpoint nor the ischemic/thromboembolic endpoint (Table 3).

**TABLE 3:** Overview of five trials comparing dual versus triple therapy in females versus males

		Endpoint of bleeding (trial definition)					
		*N*	*n*	Relative frequency	Hazard ratio (dual vs triple)	95% CI	Interaction p value
WOEST	Male	448	97	0.22	0.4	0.26–0.62	0.83
	Female	115	31	0.27	0.34	0.17–0.72	
							
PIONEER AF‐PCI	Male	1026	224	0.22	0.63	0.47–0.84	0.45
	Female	368	102	0.28	0.51	0.32–0.80	
							
RE‐DUAL PCI (dabigatran 110 mg)	Male	1478	307	0.21	0.46	0.37–0.59	0.084
	Female	484	108	0.22	0.69	0.47–1.01	
							
RE‐DUAL PCI (dabigatran 150 mg)	Male	1186	272	0.23	0.71	0.56–0.90	0.83
	Female	341	78	0.23	0.74	0.48–1.16	
							
ENTRUST‐AF PCI	Male	1126	224	0.20	0.79	0.594–1.039	0.50
	Female	384	80	0.21	0.93	0.598–1.434	
							
AUGUSTUS (apixaban vs warfarin)	Male	3228	406	0.13	0.7	0.57–0.85	0.86
	Female	1321	167	0.13	0.66	0.48–0.90	
							
AUGUSTUS (aspirin vs placebo)	Male	3231	408	0.13	1.98	1.62–2.43	0.38
	Female	1325	163	0.12	1.67	1.22–2.30	

		Endpoint of thrombosis/ischemia (trial definition)					
		*N*	*n*	Relative frequency	Hazard ratio (dual vs triple)	95% CI	Interaction p value
WOEST	Male	448	66	0.15	0.64	0.39–1.05	0.61
	Female	115	15	0.13	0.53	0.17–1.35	
							
PIONEER AF‐PCI	Male	1027	49	0.05	1.16	0.66–2.03	0.65
	Female	362	28	0.08	0.877	0.45–1.98	
							
RE‐DUAL PCI (dabigatran 110 mg)	Male	1478	214	0.14	1.16	0.89–1.52	0.73
	Female	484	66	0.14	1.05	0.65–1.70	
							
RE‐DUAL PCI (dabigatran 150 mg)	Male	1186	148	0.12	0.92	0.66–1.27	0.72
	Female	341	40	0.12	0.82	0.43–1.50	
							
ENTRUST‐AF PCI	Male	1120	71	0.06	1.02	0.641–1.631	0.76
	Female	386	24	0.06	1.14	0.520–2.502	
							
AUGUSTUS (apixaban vs warfarin)	Male	3277	217	0.07	0.95	0.72–1.23	0.86
	Female	1337	100	0.07	0.9	0.61–1.34	
							
AUGUSTUS (aspirin vs placebo)	Male	3277	217	0.07	0.9	0.69–1.18	0.80
	Female	1337	100	0.07	0.85	0.57–1.26	

*Note*: Definitions of primary bleeding and thrombotic end points as defined in each trial.^11^, ^13^, ^14^, ^15^, ^16^ Data from WOEST and ENTRUST‐AF PCI provided by Prof Jurrien ten Berg and Prof Pascal Vranckx, respectively.

Abbreviations: AF, atrial fibrillation; ASA, aspirin; CI, confidence interval; PCI, percutaneous coronary intervention.

## 4 DISCUSSION

There is growing recognition of a need to consider sex differences in both risk‐stratification and efficacy of treatments in cardiovascular disease.^3^ In addition to their under‐representation in large clinical trials, studies have shown that females have a higher likelihood of complications such as bleeding related to coronary revascularization procedures.^19^, ^20^, ^21^, ^22^ Moreover, females over the age of 75 with AF are at higher risk of stroke than males, and strokes in females with AF are more severe, have higher mortality and recovery is less complete than in males.^23^, ^24^, ^25^, ^26^ Females with coronary artery disease and AF have higher stroke risk than males, yet are treated less aggressively for primary and secondary stroke prevention than males.^27^ It is therefore crucial to delineate differences in treatment response between males and females with AF treated with anticoagulation undergoing PCI.

This subgroup analysis of the RE‐DUAL PCI trial studied potential interactions between sex and treatment with dual therapy with dabigatran and P2Y_12_ inhibition versus triple therapy with warfarin, P2Y_12_ inhibition, and aspirin specifically in the setting of patients with AF undergoing PCI. Consistent with the overall study results, we found no statistically significant interaction between sex and treatment, suggesting that both female and male patients with AF undergoing PCI benefit from a strategy of dual antithrombotic therapy with dabigatran in preference to triple therapy with warfarin. To our knowledge, this is the first dedicated subgroup analysis in the context of AF and PCI to delineate the sex‐interaction in antithrombotic treatment response. To gather a broader perspective, we tabulated the subgroup information by sex from our study and the four studies WOEST, PIONEER AF‐PCI, ENTRUST‐AF PCI, and AUGUSTUS.^13^, ^14^, ^15^, ^16^ (Table 3) In all these trials, a similar finding was seen as in RE‐DUAL PCI: there was no significant interaction between sex and treatment in the benefit see for a lower bleeding risk, nor any difference in thromboembolic events.^13^, ^14^, ^15^, ^16^

In the AUGUSTUS trial in particular, the investigators separately compared the rates of thrombotic events and bleeding between the oral anticoagulants apixaban and warfarin, and the addition of aspirin to oral anticoagulation and P2Y_12_ inhibition. In the endpoint of time to first ISTH MBE or CRNMBE between apixaban and warfarin, they found no interaction between sex and treatment in the superiority of apixaban compared to warfarin. In the efficacy endpoint of time to death or ischemic events, no difference between apixaban and warfarin was found, which held true of both males and females. AUGUSTUS also found a significant increase in bleeding without a difference in the ischemic endpoint for those treated with aspirin versus placebo in both males and females without a significant interaction between sex and treatment.^16^

All these trials all had considerably lower recruitment of females than males, with numbers comparable to that found in RE‐DUAL PCI (about 25%). However, when considered together, our analysis suggests no difference in safety and efficacy of dabigatran, and other NOACs as a class, between males and females in their use after PCI for patients with AF. The results of the overall RE‐DUAL PCI trial and indeed all the trials in this area appear to be generalizable to both females and males.

## 5 LIMITATIONS

As in any exploratory subgroup analysis, this analysis is not adequately powered, and no definitive statistical conclusion should be drawn from its results. In addition, consistent with many large cardiovascular trials, there is considerable imbalance with fewer females than males enrolled in the RE‐DUAL PCI trial which restricts definitive comparisons between the groups. Therefore, all results should be regarded as hypothesis‐generating rather than definitive evidence. However, this subgroup analysis adds to a growing body of knowledge exploring differences in sex in response to antithrombotic treatment for cardiovascular disease and will be of interest to practicing cardiologists.

## 6 CONCLUSION

This subgroup analysis of the RE‐DUAL PCI trial suggests no difference in benefit on safety and similarity on thromboembolic efficacy between females and males, supporting the use of dabigatran dual therapy over warfarin triple therapy in both sexes.

## CONFLICT OF INTEREST

David S. Eccleston and Joseph M. Kim report no conflicts of interest. Jurien M. ten Berg has received advisory/consulting/speaker fees from Accumetrics, AstraZeneca, Bayer HealthCare, Boehringer Ingelheim, Bristol‐Myers Squibb, Daiichi Sankyo, Eli Lily, Ferrer, The Medicines Company, and Pfizer, and has received research grants from AstraZeneca, ZonMw. P. Gabriel Steg has received research grants from Amarin, Bayer, Sanofi, and Servier; speaking or consulting fees from Amarin, Amgen, AstraZeneca, Bayer/Janssen, Boehringer Ingelheim, Bristol‐Myers Squibb, Idorsia, Lilly, Merck, Novartis, Novo Nordisk, Pfizer, Regeneron, Sanofi, and Servier. Deepak L. Bhatt discloses the following relationships ‐ Advisory Board: Cardax, CellProthera, Cereno Scientific, Elsevier Practice Update Cardiology, Level Ex, Medscape Cardiology, PhaseBio, PLx Pharma, Regado Biosciences; Board of Directors: Boston VA Research Institute, Society of Cardiovascular Patient Care, TobeSoft; Chair: American Heart Association Quality Oversight Committee; Data Monitoring Committees: Baim Institute for Clinical Research (formerly Harvard Clinical Research Institute, for the PORTICO trial, funded by St. Jude Medical, now Abbott), Cleveland Clinic (including for the ExCEED trial, funded by Edwards), Contego Medical (Chair, PERFORMANCE 2), Duke Clinical Research Institute, Mayo Clinic, Mount Sinai School of Medicine (for the ENVISAGE trial, funded by Daiichi Sankyo), Population Health Research Institute; Honoraria: American College of Cardiology (Senior Associate Editor, Clinical Trials and News, ACC.org; Vice‐Chair, ACC Accreditation Committee), Baim Institute for Clinical Research (formerly Harvard Clinical Research Institute; RE‐DUAL PCI clinical trial steering committee funded by Boehringer Ingelheim; AEGIS‐II executive committee funded by CSL Behring), Belvoir Publications (Editor in Chief, Harvard Heart Letter), Duke Clinical Research Institute (clinical trial steering committees, including for the PRONOUNCE trial, funded by Ferring Pharmaceuticals), HMP Global (Editor in Chief, Journal of Invasive Cardiology), Journal of the American College of Cardiology (Guest Editor; Associate Editor), K2P (Co‐Chair, interdisciplinary curriculum), Level Ex, Medtelligence/ReachMD (CME steering committees), MJH Life Sciences, Population Health Research Institute (for the COMPASS operations committee, publications committee, steering committee, and USA national co‐leader, funded by Bayer), Slack Publications (Chief Medical Editor, Cardiology Today's Intervention), Society of Cardiovascular Patient Care (Secretary/Treasurer), WebMD (CME steering committees); Other: Clinical Cardiology (Deputy Editor), NCDR‐ACTION Registry Steering Committee (Chair), VA CART Research and Publications Committee (Chair); Research Funding: Abbott, Afimmune, Amarin, Amgen, AstraZeneca, Bayer, Boehringer Ingelheim, Bristol‐Myers Squibb, Cardax, Chiesi, CSL Behring, Eisai, Ethicon, Ferring Pharmaceuticals, Forest Laboratories, Fractyl, Idorsia, Ironwood, Ischemix, Lexicon, Lilly, Medtronic, Pfizer, PhaseBio, PLx Pharma, Regeneron, Roche, Sanofi Aventis, Synaptic, The Medicines Company; Royalties: Elsevier (Editor, Cardiovascular Intervention: A Companion to Braunwald's Heart Disease); Site Co‐Investigator: Biotronik, Boston Scientific, CSI, St. Jude Medical (now Abbott), Svelte; Trustee: American College of Cardiology; Unfunded Research: FlowCo, Merck, Novo Nordisk, Takeda. Stefan H. Hohnloser has received personal fees from Bayer HealthCare, Boehringer Ingelheim, Bristol‐Myers Squibb, Daiichi Sankyo, Medtronic, Pfizer, SJM, and ZOLL. Anne de Veer reports consulting fees from Bayer and Boehringer Ingelheim. Matias Nordaby is an employee of Boehringer Ingelheim. Corinna Miede is an employee of mainanalytics GmbH, contracted by Boehringer Ingelheim International GmbH & Co. KG. Takeshi Kimura reports grants from Boehringer Ingelheim. Gregory Y. H. Lip has served as a consultant for Bayer/Janssen, Bristol‐Meyers Squibb/Pfizer, Medtronic, Boehringer Ingelheim, Novartis, Verseon, and Daiichi Sankyo. He has been a speaker for Bayer, Bristol‐Meyers Squibb/Pfizer, Boehringer Ingelheim, Daiichi Sankyo, and Medtronic. No fees are personally received. Jonas Oldgren has received fees to his institution from AstraZeneca, Bayer HealthCare, Boehringer Ingelheim, Bristol‐Myers Squibb, Daiichi Sankyo, Pfizer, Portola, Roche Diagnostics, and Sanofi. Christopher P. Cannon has received research grants Research Grants from: Amgen, Boehringer‐Ingelheim (BI), Bristol‐Myers Squibb (BMS), Daiichi Sankyo, Janssen, Merck, Novo Nordisk, Pfizer, and consulting fees from Aegerion, Alnylam, Amarin, Amgen, Applied Therapeutics, Ascendia, BI, BMS, Corvidia, Eli Lilly, HLS Therapeutics, Innovent, Janssen, Kowa, Lexicon, Merck, Pfizer, Rhoshan, Sanofi.

## Data Availability

The data that support the findings of this study are available from the corresponding author upon reasonable request.
